# MEG-PPIS: a fast protein–protein interaction site prediction method based on multi-scale graph information and equivariant graph neural network

**DOI:** 10.1093/bioinformatics/btae269

**Published:** 2024-04-18

**Authors:** Hongzhen Ding, Xue Li, Peifu Han, Xu Tian, Fengrui Jing, Shuang Wang, Tao Song, Hanjiao Fu, Na Kang

**Affiliations:** Qingdao Institute of Software, College of Computer Science and Technology, China University of Petroleum (East China), Qingdao, Shandong 266580, China; Qingdao Institute of Software, College of Computer Science and Technology, China University of Petroleum (East China), Qingdao, Shandong 266580, China; Qingdao Institute of Software, College of Computer Science and Technology, China University of Petroleum (East China), Qingdao, Shandong 266580, China; Qingdao Institute of Software, College of Computer Science and Technology, China University of Petroleum (East China), Qingdao, Shandong 266580, China; Qingdao Institute of Software, College of Computer Science and Technology, China University of Petroleum (East China), Qingdao, Shandong 266580, China; Qingdao Institute of Software, College of Computer Science and Technology, China University of Petroleum (East China), Qingdao, Shandong 266580, China; Qingdao Institute of Software, College of Computer Science and Technology, China University of Petroleum (East China), Qingdao, Shandong 266580, China; School of Humanities and Law, China University of Petroleum (East China), Qingdao, Shandong 266580, China; The Ninth Department of Health Care Administration, the Second Medical Center, Chinese PLA General Hospital, Beijing, 100853, China

## Abstract

**Motivation:**

Protein–protein interaction sites (PPIS) are crucial for deciphering protein action mechanisms and related medical research, which is the key issue in protein action research. Recent studies have shown that graph neural networks have achieved outstanding performance in predicting PPIS. However, these studies often neglect the modeling of information at different scales in the graph and the symmetry of protein molecules within three-dimensional space.

**Results:**

In response to this gap, this article proposes the MEG-PPIS approach, a PPIS prediction method based on multi-scale graph information and E(n) equivariant graph neural network (EGNN). There are two channels in MEG-PPIS: the original graph and the subgraph obtained by graph pooling. The model can iteratively update the features of the original graph and subgraph through the weight-sharing EGNN. Subsequently, the max-pooling operation aggregates the updated features of the original graph and subgraph. Ultimately, the model feeds node features into the prediction layer to obtain prediction results. Comparative assessments against other methods on benchmark datasets reveal that MEG-PPIS achieves optimal performance across all evaluation metrics and gets the fastest runtime. Furthermore, specific case studies demonstrate that our method can predict more true positive and true negative sites than the current best method, proving that our model achieves better performance in the PPIS prediction task.

**Availability and implementation:**

The data and code are available at https://github.com/dhz234/MEG-PPIS.git.

## 1 Introduction

The study of protein–protein interaction stands as a pivotal focus within biological research, wielding significant influence across diverse biological processes including cell signaling, metabolic regulation, and the cell cycle. Understanding the intricate interactions among proteins can unveil complex molecular networks within cells (Li et al., 2020), offer insights into disease mechanisms (Richards *et al.*, 2021), and pave the way for innovation in drug development (Lu *et al.*, 2020). An important related issue in the study of protein–protein interaction is the identification of protein–protein interaction sites (PPIS). Delving into an exhaustive exploration of these interaction sites enables a deeper understanding of protein interaction mechanisms, thereby furnishing crucial insights for disease treatments and the creation of novel drugs (Ezkurdia *et al.*, 2009, Aumentado-Armstrong *et al.*, 2015). However, biologically experimental methods, such as X-ray crystallography and two-hybrid screening to identify PPIS, are time-consuming and costly (Shoemaker and Panchenko, 2007). Developing fast and convenient computational methods to identify PPIS has become one of the key issues in protein interaction research.

Early computational methods for PPIS prediction were based on machine learning methods, such as Naive Bayes classifier, random forest, and XGBoost (Murakami and Mizuguchi, 2010, Northey *et al.*, 2018, Wang et al., 2021; Zhang and Kurgan, 2019, Deng *et al.*, 2020). These methods selected appropriate feature methods to represent proteins through feature engineering, and then learn useful information from the features for prediction through machine learning algorithms. In recent years, plenty of researchers have begun to focus on deep learning algorithms such as Convolutional Neural Network (CNN), Recurrent Neural Network (RNN), and Graph Neural Network (GNN) to perform PPIS prediction tasks, which have achieved good results.

Methods based on CNN and RNN take proteins as one-dimensional amino acid sequences and learn information about proteins on the sequences. CNN-based methods can effectively capture local patterns in protein sequences through convolution operations. And RNN-based methods provide a more robust characterization of long-range correlations in protein sequences because they can handle long-range dependencies and global information from the sequence. For example, DeepPPISP (Zeng *et al.*, 2020) used TextCNN to extract global features from the protein sequence. It integrated these global features with local features within the network for prediction, leading to good outcomes. A simplified long short-term memory (SLSTM) network was used in DLPred (Zhang *et al.*, 2019) to design a PPIS prediction model. DELPHI (Li *et al.*, 2021) designed an integrated method that combines CNN and RNN with fine-tuning technology in the model architecture to make full use of the protein sequence information.

Unlike CNN and RNN methods, which concentrate on protein sequences to extract protein information, GNN-based methods construct protein graphs using three-dimensional conformational information of proteins. This enables a more precise capture of the structural characteristics of proteins. The capability of protein graph representation to model tertiary structure information effectively has led to notable advances with graph neural network methods. For example, GraphPPIS (Yuan *et al.*, 2022) utilized a deep graph convolutional neural network (GCN) framework for PPIS prediction, and its effect significantly improved compared to sequence-based methods. Residue-based graph attention and convolutional network (RGN) combined GCN and graph attention networks (GAT), utilizing a deep residual structure to further extract deeper protein features (Wang *et al.*, 2022). DeepProSite (Fang *et al.*, 2023) was a topology-aware model based on Graph Transformer. It used the prior knowledge of the protein language model and combined the three-dimensional structural information to achieve an accurate prediction of PPIS. AGAT-PPIS (Zhou *et al.*, 2023) improved GAT and can use weighted neighborhood node features and neighborhood edge features to update node embeddings, thus integrating more protein structure information.

Although current GNN-based protein site prediction methods such as AGAT-PPIS have achieved good results, there are still two aspects worth improving in GNN-based PPIS prediction methods. First, many studies have shown that multi-scale feature learning strategies on protein feature matrices have achieved good improvements in downstream tasks such as protein–protein interaction prediction and PPIS prediction (Zeng *et al.*, 2020, Li *et al.*, 2023). However, current graph-based PPIS prediction methods ignored the multi-scale modeling of information on the protein graph. Second, the conformational characteristics of protein molecules in three-dimensional space significantly influence protein–protein interactions. Studies on molecule representation learning indicated that incorporating models accounting for the spatial equivariant properties of molecules could enhance prediction results (Han *et al.*, 2022). However, existing graph-based protein site prediction methods often neglected the inherent symmetries occurring in space during protein–protein interactions.

In this article, to improve the existing GNN-based PPIS methods, we proposed a PPIS prediction model based on multi-scale graph information and equivariant graph neural networks (MEG-PPIS). Our MEG-PPIS model adopted a weight-sharing strategy in the feature update network on the original graph and the subgraph divided by the graph pooling method. This allowed the model to consider two different ranges of neighbor messages on the original graph and subgraph for the same node when aggregating node features, thus learning the different scale patterns of the protein graphs and enhancing the learning ability of the model. Additionally, to enable the model to learn the spatial equivariance in the original protein graph and subgraph, MEG-PPIS used EGNN as the graph network layer, ensuring that the learning of protein molecular features conformed to equivariances such as rotation, reflection, and translation in three-dimensional space. Furthermore, the residual connection between graph update layers helped our model mitigate the over-smoothing problem. Experimental results demonstrated that our model outperformed other advanced PPIS prediction methods in seven indicators in four test sets. Compared to the state-of-the-art (SOTA) model AGAT-PPIS, our model achieved more accurate predictions for specific protein cases. Furthermore, the average prediction time of our model across the four test sets was roughly reduced to 20% of that of AGAT-PPIS, signifying a higher prediction efficiency.

## 2 Materials and methods

### 2.1 Datasets

In this study, benchmark datasets were the same as those in the previous work AGAT-PPIS (Zhou *et al.*, 2023), including training set (Train_335-1) and testing sets (Test_60, Test_315-28, Ubtest_31-6).

During model comparison, Btest_31-6 was constructed using the monomeric structures of the 25 protein complex structures in Test 60 corresponding to UBtest_31-6. Regarding datasets utilization, training for our approach in this study was carried out on the Train_335-1 dataset. Other datasets were employed to assess model performance, with Test_60 serving primarily as the benchmark test set for performance comparison, while the remaining datasets were used to evaluate the generalization ability.

The AGAT-PPIS datasets were obtained by fine-tuning the GraphPPIS datasets (Yuan *et al.*, 2022), and proteins inconsistent with the sequences on the PDB website were removed from the GraphPPIS datasets. The specific information of the GraphPPIS datasets and AGAT-PPIS datasets is shown in Supplementary Tables S1 and S2.

### 2.2 Protein representation

In this study, we employed an undirected graph (G, V) to characterize proteins. The graph nodes represent amino acids, while the edges represent connections between these amino acids. Based on the sequence and structure information of the protein, we obtained the node feature matrix X and the node adjacency matrix A corresponding to the protein graph and finally constructed a graph representation of the protein according to the rules established by the graph. Regarding the details, we gave a detailed introduction in the following.

Node features were acquired through the processing of both protein sequence and protein structure information. The processing method was referred to AGAT-PPIS (Zhou *et al.*, 2023). Features based on protein sequence information included position-specific scoring matrix (PSSM) and hidden Markov model matrix (HMM). The PSSM was generated using PSI-BLAST (Altschul *et al.*, 1997) by comparing the input protein sequence with the sequences in the UniRef 90 database. It reflects amino acid distribution probabilities, indicating evolutionary information about proteins. The HMM was constructed using HHblits v3.0.3 (Remmert *et al.*, 2012) with default parameters to query the UniClust30 database, representing amino acid insertion and deletion details. Features based on protein structural information included definition of secondary structure of proteins (DSSP), atomic features (AF), and position embedding features (PEF). The DSSP matrix was created using the protein’s 3D structure through the DSSP program (Kabsch and Sander, 1983). Each amino acid corresponded to a 14-dimensional vector within the DSSP matrix, encompassing one-hot encoding of the secondary structure state, relative solvent-accessible surface area, and sine/cosine values of the peptide chain backbone torsion angles. The atomic features of each atom on the residue (excluding hydrogen atoms) included seven characteristics: atomic mass, B factor, whether it was a residue side chain atom, electron charge, the number of hydrogen atoms bonded to it, whether it was part of a ring, and the van der Waals radius of the atom. The position embedding features of the residue described the position information of the residue. We used the amino acid residue side chain centroid (SC) to represent the residue coordinates and perform the position embedding representation of the residue. The final node feature matrix can be expressed as an L*62 feature matrix, where L is the number of amino acids in the protein chain, and 62 represents the feature dimension.

(1) $$X=[X_{\mathrm{PSSM}},X_{\mathrm{HMM}},X_{\mathrm{DSSP}},X_{AF},X_{\mathrm{PEF}}]$$

In Equation (1), *X_PSSM_*, *X_HMM_*, and *X_DSSP_* are the PSSM, HMM, and DSSP feature matrices, respectively. *X_AF_* is the atomic feature matrix of the amino acid in the protein. *X_PEF_* is the position feature matrix of the amino acid in the protein.

In our study, edge features focused solely on spatial relationships among amino acid nodes within the protein graph representation. We derived amino acid positional data from the protein’s PDB file and computed the Euclidean distance between all amino acids. Using a cutoff distance, we determined whether the distance between two amino acids satisfied the required relationship criterion. If the cutoff value was below, an edge was established between the respective amino acid nodes; otherwise, no edge was created. This process yielded an adjacency matrix, with a value of 1 denoting an edge between nodes and 0 indicating no edge. In alignment with prior research (Zhou *et al.*, 2023), we set the hyperparameter cutoff distance at 14 Å.

### 2.3 Model overview

**Figure 1.**
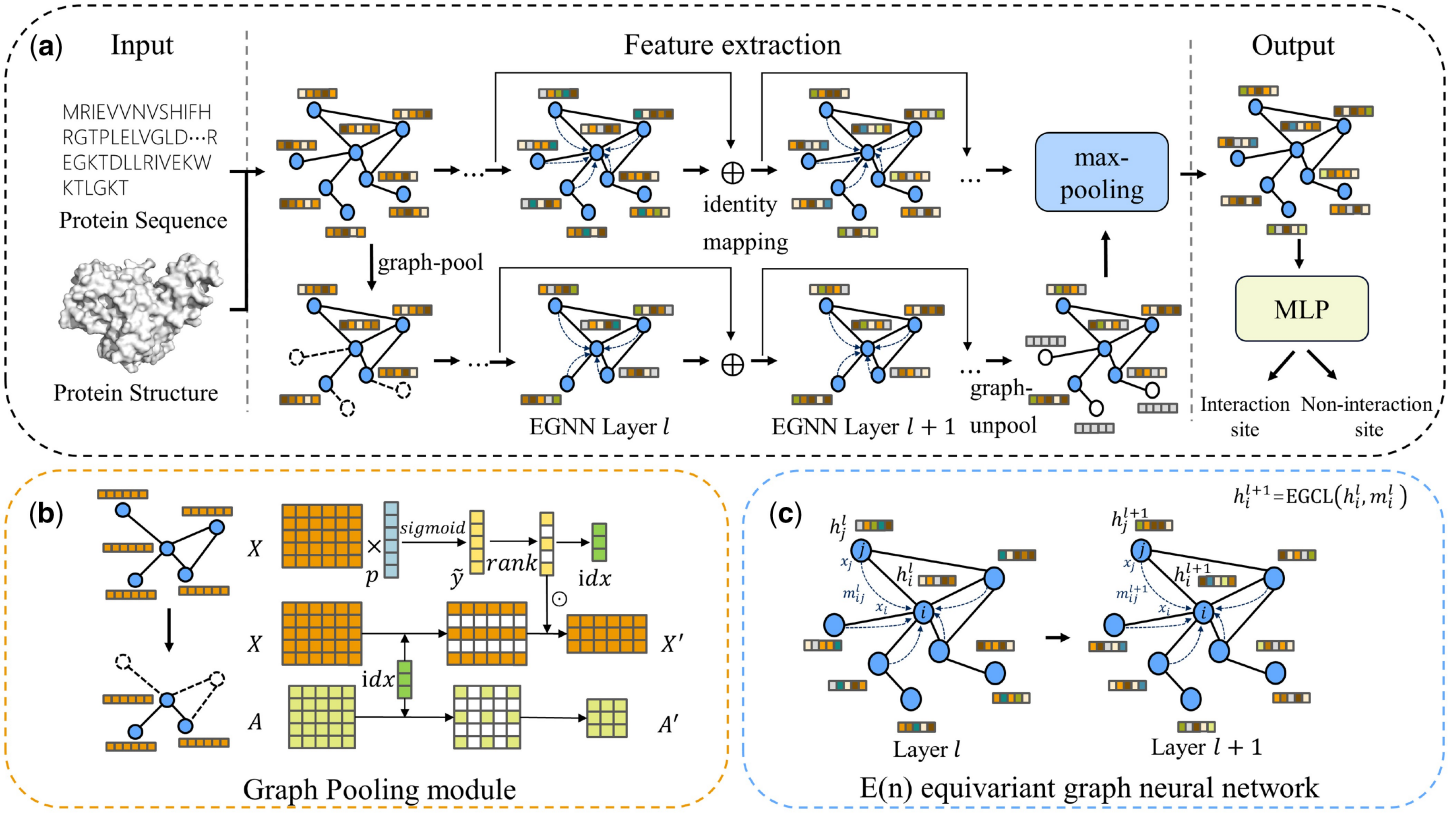
The overall architecture of MEG-PPIS. (a) Three parts of the MEG-PPIS model. (b) Graph pooling module. (c) E(n) equivariant graph neural network.

The overall architecture of the MEG-PPIS model proposed is shown in Fig. 1. The MEG-PPIS model consists of three parts: input, feature extraction, and output (Fig. 1a). First, we extracted node and edge features from the protein sequence and structure information and constructed a graph representation for the protein. In the feature extraction part, alongside the original graph channel, the model divided the original protein graph into subgraph as another channel through the graph pooling method (Fig. 1b). We fed the node features and edge features of the protein into the E(n) equivariant graph neural network (Fig. 1c) on both the original graph and subgraph to conduct aggregated updates of node embeddings. The network weights were shared during the update process for both the original graph and subgraph. Following the features update, a graph unpooling operation was applied to restore the subgraph to its original shape. Subsequently, a maximum pooling operation was performed on both the original graph and subgraph to synthesize the corresponding node features. In the final step, the model’s output part underwent feature dimensionality reduction through three linear layers, yielding the ultimate prediction result. Strategies such as graph pooling, graph unpooling, and weight-sharing facilitated the model in learning two approaches to aggregate node neighborhood messages on both the original graph and subgraph. The E(n) equivariant graph neural network guaranteed the equivariance of protein molecules throughout the feature aggregation process. At the same time, to alleviate the over-smoothing problem, we used the identity mapping residual structure at each layer of the graph node feature update.

### 2.4 Graph pooling module and graph unpooling module

CNN-based multi-scale learning used convolution kernels of different sizes to model different scale features of the protein (Zeng *et al.*, 2020, Li *et al.*, 2023). Regarding the graph structure, we took inspiration from the Graph-Unet approach (Gao and Ji, 2019) and devised the graph pooling module to obtain the subgraph, enabling subsequent graph update networks to learn patterns of different scales on both the original graph and subgraph. The graph pooling module partitioned the subgraph through low-dimensional mapping and sorting based on the node feature matrix of the original graph. It then selected and acquired the node feature matrix X′ and adjacency matrix A′ of the subgraph.

(2) y=Xp/||p||

(3) $$\tilde{y}=\mathrm{sigmoid}(y)$$

(4) $$\mathrm{idx}=\mathrm{rank}(\tilde{y},k)$$

(5) $$\tilde{X}=X(\mathrm{idx},:)$$

(6) $$A^{\prime }=A^{2}(\mathrm{idx},\mathrm{idx})$$

(7) $$X^{\prime }=\tilde{X}⊙\left(\tilde{y}1_{C}^{T}\right)$$

The node feature matrix *X* and the mapping vector *p* are multiplied to obtain *y*, and then a sigmoid function is performed on *y* to obtain the node scores $\tilde{y}$ [Equations (2)–(3)]. The $\mathrm{rank}(\tilde{y},k)$ is an operation that sorts nodes by the scores and returns the indices of the *k* largest values in $\tilde{y}$ as idx. The idx contains the indices of the nodes selected for the subgraph [Equation (4)]. X(idx,:) and $A^{2}(\mathrm{idx},\mathrm{idx})$ are extracted from the original feature matrix and adjacency matrix according to the idx to form the node feature matrix $\tilde{X}$ and adjacency matrix A′ of the subgraph [Equation (5)–(6)]. The notation $1_{C}^{T}$ denotes a vector of size *C* with all components equal to 1. The final node feature matrix of the subgraph X′ is updated by performing the element-wise matrix product of $\tilde{X}$ and $\tilde{y}1_{C}^{T}$ [Equation (7)]. During the node selection process, the mapping vector *p* is learnable, allowing for the training-based learning of node selection within the subgraph. The operation described in Equation (7) ensures that this process is trainable through gradient updates.

At the same time, to facilitate feature fusion with the node features learned from the original graph, the subgraph was restored to the size of the original graph through an inverse operation after the features on the subgraph were updated [Equation (8)].

(8) $$X^{\prime \prime }=\text{distribute}\;\left(0_{N\times C},X^{\prime },\mathrm{idx}\right)$$

In Equation (8), the operation $\;\text{distribute}\;\left(0_{N\times C},X^{\prime },\mathrm{idx}\right)$ involves updating the node feature matrix of the subgraph X′ into the initially empty feature matrix $0_{N\times C}$ of the new graph based on the idx. Finally, the new graph X″ is consistent with the shape of the original graph, the row vectors with indexes in X″ are the same as the row vectors in X′.

### 2.5 Graph node feature update layer

In this study, the feature update method in the protein graph adopted the E(n) equivariant graph neural network (EGNN) (Satorras *et al.*, 2021) architecture. The network architecture consisted of a stack of equivariant graph convolutional layers (EGCL). EGCL updated the node coordinates and node features of the current layer by aggregating edge information, as well as node coordinates and features from the previous layer. Compared with the update method of traditional graph convolutional neural network, the EGCL introduced coordinate update during node feature aggregation for equivariant message transmission. The updated equations are as follows.

(9) $$m_{ij}^{l}=\varphi_{e}\left(h_{i}^{l},h_{j}^{l},{\left\|x_{i}^{l}-x_{j}^{l}\right\|}^{2},a_{ij}\right)$$

(10) $$x_{i}^{l+1}=x_{i}^{l}+C\sum_{j\neq i}\left(x_{i}^{l}-x_{j}^{l}\right)\varphi_{x}\left(m_{ij}^{l}\right)$$

(11) $$m_{i}^{l}=\sum_{j\neq i}\left(m_{ij}^{l}\right)$$

(12) $$h_{i}^{l+1}=\varphi_{h}\left(h_{i}^{l},m_{i}^{l}\right)$$

In the above equations, $m_{ij}^{l}$ describes the message delivered between node i and node j in layer l. $m_{ij}^{l}$ is obtained by transforming the node i feature $h_{i}^{l}$, the node j feature $h_{j}^{l}$, the relative square distance ${\left\|x_{i}^{l}-x_{j}^{l}\right\|}^{2}$between the coordinates of node i and node j, and the edge feature $a_{ij}$ through an edge operation $\varphi_{e}$ [Equation (9)]. The update of the coordinate for node i is computed through a weighted sum of the differences in coordinate embeddings from the previous layer. This sum is then normalized using the factor C = 1/(M-1), where M represents the number of nodes in the graph [Equation (10)]. Then we aggregate the messages passed between node i and all its neighbor nodes to obtain $m_{i}^{l}$[Equation (11)]. And node operation $\varphi_{h}$ is performed on $m_{i}^{l}$ and the feature $h_{i}^{l}$ of node i in layer l to obtain the updated feature $h_{i}^{l+1}$ of node i in layer l + 1[Equation (12)]. Edge operations $\varphi_{e}$, coordinate operations $\varphi_{x}$, and node operations $\varphi_{h}$ are composed of multi-layer perceptions and nonlinear operations.

### 2.6 Residual identity mapping and max pooling aggregation

When the number of layers in a graph neural network deepens, an over-smoothing phenomenon often occurs, where the features of each node on the graph tend to become overly consistent. To mitigate this over-smoothing issue, the model incorporated identity mapping connections between the multi-layer EGCL modules, as denoted in Equation (13). The implementation of residual connection operations allowed for an expanded number of model layers, facilitating the acquisition of deep-seated node features within the graph. Concurrently, following the acquisition and aggregation of graph features across various scales, the model consolidated these diverse features from distinct graphs through maximum pooling, as shown in Equation (14).

(13) $$h^{l+1}=\mathrm{relu}\left(h^{l}+\mathrm{EGCL}\left(h^{l},m^{l}\right)\right)$$

(14) $$h_{\text{aggregated}}=\underset{g\in G}{\max}\left(h_{g}\right)$$

In Equation (13), the node features of layer l and layer l + 1 are, respectively, expressed as *h^l^* and $h^{l+1}$.$\mathrm{EGCL}\left(h^{l},m^{l}\right)$ represents the features updated by the EGCL module. We add it to the features before updating *h^l^* and then pass it through a relu activation function to finally get $h^{l+1}$. In Equation (14), a maximum pooling operation is used to obtain the maximum value of the corresponding node on the origin graph and subgraph, and finally the aggregated feature *h_aggregated_* is obtained.

## 3 Results and discussion

### 3.1 Experimental setup

In this study, we used the PyTorch framework to build the model. Specifically, the cutoff distance for edge representation in the protein graph was set to 14 Å. The division ratio of the subgraph in the graph pooling module was configured at 0.6, and the number of EGCL layers was set to 6. The EGCL layer number and subgraph partition ratio hyperparameters were determined through experimentation, with detailed results provided in Supplementary Tables S3–S4. The feature embedding dimension was aligned with the initial input feature dimension. The output dimensions of the prediction layer network were 20, 10, and 2, respectively. Model training employed a learning rate of 0.001 over 50 epochs.

During training, the cross-entropy(CE) loss function guided gradient updates [Equation (15)]. In Equation (15), *n* is the number of samples, *m* is the number of classes, $y_{i,c}$ represents the true label of the sample, and $p_{i,c}$ represents the corresponding predicted value. The Adam optimizer was employed for model optimization, and the ReduceLROnPlateau scheduler dynamically adjusted the learning rate throughout the training process. The evaluation metrics align with those of AGAT-PPIS (See Supplementary for additional details).

(15) $$\;\text{CE loss}=-\frac{1}{n}\sum_{i=1}^{n}\sum_{c=1}^{m}y_{i,c}\;\text{log}\;\left(p_{i,c}\right)$$

### 3.2 Performance comparison with other methods

On the independent test set Test_60, we performed a comparative analysis of the performance between MEG-PPIS and other models designed for predicting PPIS. As shown in Table 1, MEG-PPIS performs better than other models on the seven evaluation indicators. Compared with five methods based on protein sequence information [PSIVER (Murakami and Mizuguchi, 2010), ProNA2020 (Qiu *et al.*, 2020), SCRIBER (Zhang and Kurgan, 2019), DLPred (Zhang *et al.*, 2019), and DELPHI (Li *et al.*, 2021)], our method greatly improves the performance of PPIS prediction due to the introduction of structural information. Compared with the four GNN-based methods [GraphPPIS (Yuan *et al.*, 2022), RGN (Wang *et al.*, 2022), DeepProSite (Fang *et al.*, 2023), AGAT-PPIS (Zhou *et al.*, 2023)] and three methods [DeepPPISP (Zeng *et al.*, 2020), SPPIDER (Porollo and Meller, 2007), MaSIF-site (Gainza *et al.*, 2020)] that consider structural information but do not use GNN model, our model has different degrees of improvement in various indicators. Compared with the current best-performing model AGAT-PPIS, our model outperforms it in all indicators. Numerically, our model improves ACC by 0.022, Precision by 0.066, Recall by 0.054, F1 by 0.061, MCC by 0.074, AUROC by 0.025, and AUPRC by 0.092. Among them, the MCC and AUPRC indicators have improved significantly, increasing by 15.29% and 16.03% compared with AGAT-PPIS. We also plotted the ROC curves and PR curves of the two models on Test_60, as shown in Supplementary Fig. S1.

**Table 1.** Performance comparison with other models on Test_60.

Method	ACC	Precision	Recall	F1	MCC	AUROC	AUPRC
PSIVER	0.561	0.188	0.534	0.278	0.074	0.573	0.190
ProNA2020	0.738	0.275	0.402	0.326	0.176	N/A	N/A
SCRIBER	0.667	0.253	0.568	0.350	0.193	0.665	0.278
DLPred	0.682	0.264	0.565	0.360	0.208	0.677	0.294
DELPHI	0.697	0.276	0.568	0.372	0.225	0.699	0.319
DeepPPISP	0.657	0.243	0.539	0.335	0.167	0.653	0.276
SPPIDER	0.752	0.331	0.557	0.415	0.285	0.755	0.373
MaSIF-site	0.780	0.370	0.561	0.446	0.326	0.775	0.439
GraphPPIS	0.776	0.368	0.584	0.451	0.333	0.786	0.429
RGN	0.785	0.382	0.587	0.463	0.349	0.791	0.441
DeepProSite	0.842	0.501	0.443	0.470	0.379	0.813	0.490
AGAT-PPIS	0.856	0.539	0.603	0.569	0.484	0.867	0.574
MEG-PPIS	**0.878**	**0.605**	**0.657**	**0.630**	**0.558**	**0.892**	**0.666**

*Note:* The highlighted values in bold indicate the best performance on the corresponding indicators in the table.

Furthermore, we conducted a comparative analysis of our model and AGAT-PPIS on three independent test sets: Test_315-28, Btest_31-6, and UBtest_31-6. As shown in Table 2, the results show that our model has significant improvements over AGAT-PPIS in the MCC and AUPRC indicators of three independent test sets. Comparing the effects of three independent test sets, we can intuitively see that our model has stronger generalization and robustness than AGAT-PPIS.

**Table 2.** Performance comparison of MEG-PPIS and AGAT-PPIS on the Test_315-28, BTest_31-6 and UBtest_31-6.

Method	Test_315-28		BTest_31-6		UBtest_31-6	
	MCC	AUPRC	MCC	AUPRC	MCC	AUPRC
AGAT-PPIS	0.481	0.572	0.485	0.583	0.327	0.365
MEG-PPIS	**0.557**	**0.651**	**0.583**	**0.641**	**0.356**	**0.396**

*Note:* The highlighted values in bold indicate the best performance on the corresponding indicators in the table.

### 3.3 Feature ablation experiment

In current related research work, the importance of protein sequence information features such as PSSM and HMM has been widely proven (Wang *et al.*, 2022, Yuan *et al.*, 2022). In this work, we conducted feature ablation experiments to compare the impact of protein structural information features including DSSP, atomic features (AF) and position embedding features (PEF) on model performance to guide the final feature selection of our model. In the experiment, we used only the sequence information features (PSSM+HMM) as the comparison benchmark, and gradually added more structural information features (DSSP, AF, PEF) to compare the effects. As shown in Table 3, with the addition of protein structure information features, the model has improved to varying degrees in each evaluation index. Taking the AUROC and AUPRC indicators as an example, after adding the DSSP feature, the AUROC and AUPRC indicators of the independent test set Test_60 increase by 0.026 and 0.034, respectively. After further adding AF features, the AUROC and AUPRC indicators of the independent test set Test_60 continue to increase by 0.023 and 0.046 respectively. Finally, when using all features (PSSM+HMM+DSSP+AF+PEF), the model achieves the best results of 0.892 and 0.666 on the AUROC and AUPRC indicators. Experiments show that the introduction of protein structural features can help improve the effect of the PPIS prediction model. We also conducted performance testing using only the structural feature group (DSSP+AF+PEF). The experimental results indicate that models using only the sequence feature group or the structural feature group perform similarly, but both are inferior to using both sequence and structural features simultaneously.

**Table 3.** Performance comparison of MEG-PPIS with different feature groups on independent test set Test_60.

Feature group	ACC	Precision	Recall	F1	MCC	AUROC	AUPRC
PSSM+HMM	0.829	0.469	0.623	0.535	0.440	0.836	0.550
DSSP+AF+	0.843	0.502	0.579	0.538	0.445	0.843	0.547
PEF							
PSSM+HMM+	0.841	0.498	0.652	0.565	0.476	0.862	0.584
DSSP							
PSSM+HMM+	0.873	0.597	0.612	0.604	0.529	0.885	0.630
DSSP+AF							
PSSM+HMM+	**0.878**	**0.605**	**0.657**	**0.630**	**0.558**	**0.892**	**0.666**
DSSP+AF+							
PEF							

*Note:* The highlighted values in bold indicate the best performance on the corresponding indicators in the table.

### 3.4 Model architecture analysis experiment

Inspired by pooling operations in one-dimensional sequences and two-dimensional images for multi-scale learning, we incorporated a graph pooling module into the MEG-PPIS model. MEG-PPIS can learn neighborhood information aggregation methods at different scales on the original graph and the subgraph obtained by graph pooling. In this experiment, we explored the impact of the subgraph channel on the performance of our model, demonstrating the effectiveness of multi-scale learning on graphs. We comprehensively compared the effects of the model after removing the subgraph channel and the full model through ablation experiments on all test sets. As shown in Table 4, after removing the subgraph channel, the model has a certain degree of decline in different indicators on the independent test set Test_60, with a decrease of 0.029 and 0.028 in MCC and AUPRC, respectively. Further, we tested the performance of the model after removing subgraph channels in three independent test sets Test_315-28, Btest_31-6 and UBtest_31-6. As shown in Supplementary Table S5, after removing the subgraph channel, the effects on the Test_315-28 and Btest_31-6 also drop significantly. These comparison results prove that comprehensive consideration of multi-scale protein graph information through subgraph channel can improve the model effect and enhance the generalization ability of the model on different datasets to a certain extent.

**Table 4.** Performance comparison of MEG-PPIS with or without subgraph channel on Test_60.

Method	ACC	Precision	Recall	F1	MCC	AUROC	AUPRC
MEG-PPIS (without subgraph channel)	0.870	0.579	0.637	0.607	0.529	0.891	0.638
MEG-PPIS	**0.878**	**0.605**	**0.657**	**0.630**	**0.558**	**0.892**	**0.666**

*Note:* The highlighted values in bold indicate the best performance on the corresponding indicators in the table.

Considering the priori symmetry of the transformation of the properties of protein molecules in space during the binding process, we use EGNN as the graph update network of our model to effectively learn this property. Here, we verify the effectiveness of the EGNN to the model through comparative experiments. To avoid the impact of the subgraph module on the effect, we removed the subgraph channel in the comparison. At this time, our model degenerated into using EGNN on the original protein graph to update the graph information, without considering the subgraph information. We named this model EGNN-PPIS. We compared the effect of this model with the previous models that also only updated information on the original graph, proving the effectiveness of EGNN on the PPIS problem compared with other graph update networks. We selected GCN-PPIS, GAT-PPIS and AGAT-PPIS (Zhou *et al.*, 2023) for performance comparison with our model on Test_60 and Test_315-28. The compared model graph network layers used GCN, GAT and AGAT (GAT containing edge enhancement), respectively. The results in Table 5 prove that when only using EGNN to update the network on the original graph, our model performance comprehensively surpasses the comparative model in all four indicators of the two datasets. This shows that the equivariant properties of protein molecules in space in translation, rotation, and reflection have a significant impact on the PPIS problem. It guides us to consider the spatial equivariance of protein molecules when designing models.

**Table 5.** Performance comparison of EGNN-PPIS with other graph update network models on Test_60 and Test_315-28.

Method	Test_60				Test_315-28			
	F1	MCC	AUROC	AUPRC	F1	MCC	AUROC	AUPRC
GCN-PPIS	0.537	0.444	0.848	0.549	0.538	0.455	0.862	0.548
GAT-PPIS	0.547	0.455	0.843	0.563	0.546	0.467	0.864	0.556
AGAT-PPIS	0.569	0.484	0.867	0.574	0.559	0.481	0.874	0.572
EGNN-PPIS	**0.607**	**0.529**	**0.891**	**0.638**	**0.600**	**0.530**	**0.896**	**0.613**

Note: The highlighted values in bold indicate the best performance on the corresponding indicators in the table.

### 3.5 Case study

We performed a specific case study to evaluate the predictive ability of the MEG-PPIS model for specific protein chains. The results of predicting PPIS of protein 2v9t (PDB ID) chain A and protein 4kbm (PDB ID) chain B from Test_60by our model and the AGAT-PPIS model are shown in Table 6. The results show that compared to the AGAT-PPIS model, our model predicts a greater number of true positive sites and true negative sites, while also predicting fewer false positive sites and false negative sites. As shown in Supplementary Fig. S2, we also visualized the prediction results. By observing the colored part, we can find that MEG-PPIS has significantly fewer associated false positive sites in the prediction results, indicating that our model can better identify PPIS. More protein examples are provided in Supplementary Figs. S3–S5 and Supplementary Tables S6–S8.

**Table 6.** Prediction results of MEG-PPIS and AGAT-PPIS on specific proteins.

Protein	Model	TP	TN	FP	FN
2v9t, chain A	AGAT-PPIS	13	56	28	10
	MEG-PPIS	**14**	**70**	**14**	**9**
4kbm, chain B	AGAT-PPIS	12	97	34	5
	MEG-PPIS	**17**	**114**	**17**	**0**

Note: The highlighted values in bold indicate the best performance on the corresponding indicators in the table.

### 3.6 Running time analysis

**Figure 2.**
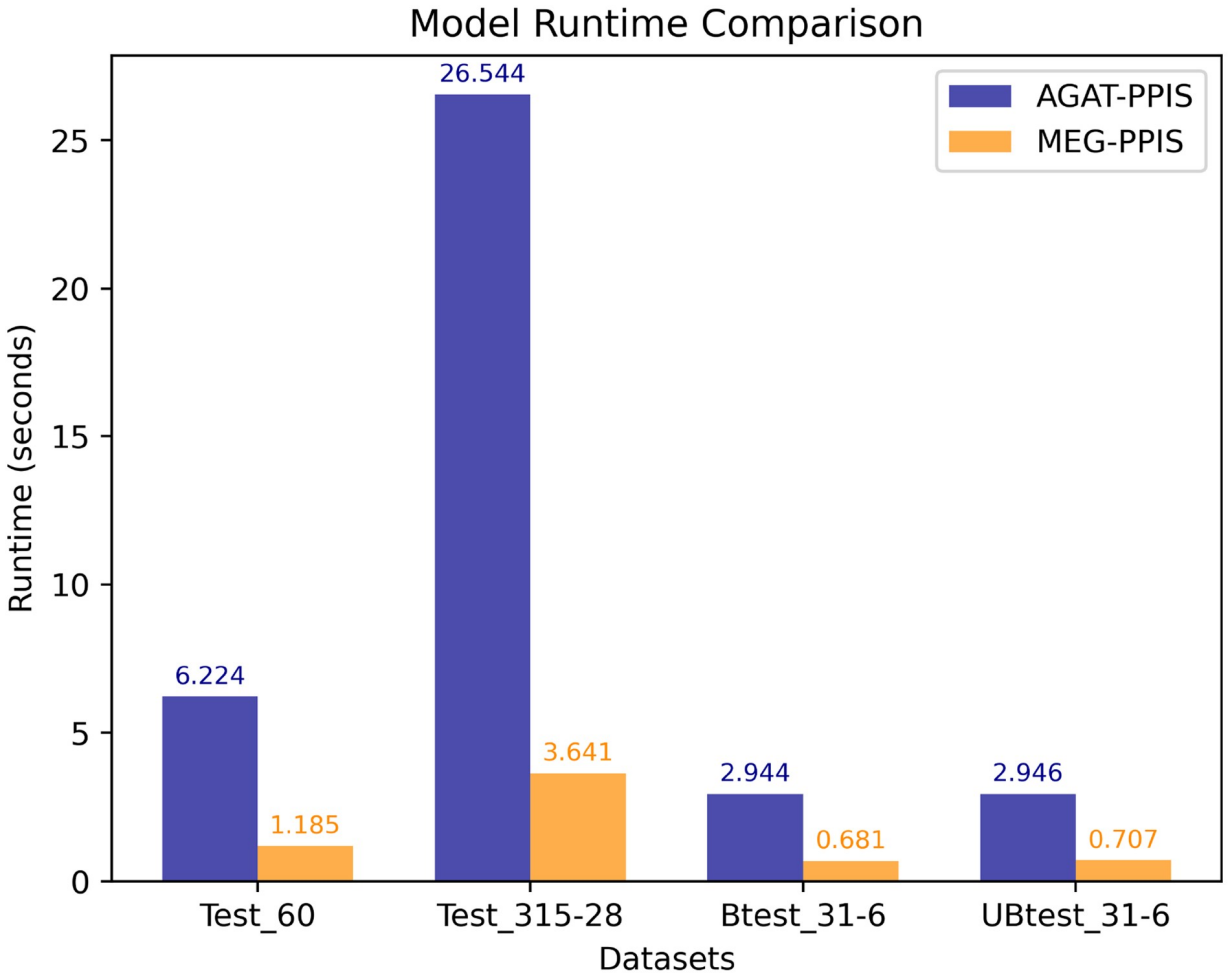
The running time of MEG-PPIS and AGAT-PPIS on different datasets

In the time comparison analysis experiment, we compared the running time of predictions between our model and the AGAT-PPIS model on different datasets. The experiments were conducted under the same hardware configuration and operating system. We ran MEG-PPIS and AGAT-PPIS 5 times on four test datasets, respectively, and averaged the five running times for prediction on each dataset. As shown in Fig. 2, the results show that the prediction time of our model is much shorter than that of AGAT-PPIS on all datasets. Especially on the Test_315-28 with a large amount of protein data, the prediction time of our model is 13.7% of the prediction time of the AGAT-PPIS model. It shows that our model has greatly improved the prediction speed and has higher prediction efficiency. We think that the higher efficiency of MEG-PPIS compared to AGAT-PPIS may mainly lie in the absence of a complex attention mechanism. Attention mechanisms require additional computation to assign attention weights during neighbor message aggregation. Additionally, AGAT-PPIS introduces extra edge features, increasing the computational complexity of the model.

## 4. Conclusion

This article introduces MEG-PPIS, an advanced model for PPIS prediction. MEG-PPIS implements protein learning at different scales on the original graph and the subgraph obtained by graph pooling through weight-sharing EGNN. EGNN maintains the spatial equivariance of molecules during the feature learning process. Comparative experimental analyses conclusively demonstrate MEG-PPIS’s superior performance over existing models. Furthermore, through ablation experiments, we underscore the significance of multi-scale learning and equivariance considerations, affirming the substantial impact of enriched protein structural features on model efficacy. Time comparison experiments further establish our model’s efficiency compared to the SOTA model. Future endeavors will concentrate on predictive analyses targeting specific protein action sites, such as antibodies or enzymes, fostering deeper insights and advancements in this domain.

## Supplementary Material

btae269_Supplementary_Data

## Data Availability

The data and code are available at https://github.com/dhz234/MEG-PPIS.git
