# Supplementary material for: MEG-PPIS: a fast protein–protein interaction site prediction method based on multi-scale graph information and equivariant graph neural network
Source: Bioinformatics. 2024 Apr 18;40(5):btae269. doi: 10.1093/bioinformatics/btae269 (PMC11252844; doi:10.1093/bioinformatics/btae269)
Supplement: btae269_Supplementary_Data [file btae269_supplementary_data.docx]

**The statistical details of datasets**

Table S1. The statistical details of GraphPPIS datasets

| Dataset | Protein chains | Interacting residues | Non-interacting residues | Proportion of interaction  site (%) |
| --- | --- | --- | --- | --- |
| Train_335 | 335 | 10374 | 55992 | 15.6 |
| Test_60 | 60 | 2075 | 11069 | 15.8 |
| Test_315 | 315 | 9355 | 55976 | 14.3 |
| Ubtest_31 | 31 | 841 | 5813 | 12.6 |

Table S2. The statistical details of AGAT-PPIS datasets

| Dataset | Protein chains | Abnormal Protein Chains | Interacting residues | Non-interacting residues | Proportion of interaction  site (%) |
| --- | --- | --- | --- | --- | --- |
| Train_335-1 | 334 | 1 | 10336 | 55872 | 15.6 |
| Test_60 | 60 | 0 | 2075 | 11069 | 15.8 |
| Test_315-28 | 287 | 28 | 8566 | 51810 | 14.2 |
| Ubtest_31-6 | 25 | 6 | 711 | 5206 | 12.0 |

**Evaluation metrics**

The task of protein site prediction can be regarded as a binary classification problem of learning protein features based on the model and then predicting whether the amino acids on the protein are binding sites.

For the model verification evaluation method, based on the evaluation index of the two-class model, we selected Accuracy (number of data correctly classified by the algorithm/number of data input to the algorithm),Precision (the proportion of positive samples predicted to all positive samples),Recall(the proportion of correctly predicted data in the total sample),F1(an indicator of precision and recall, the harmonic mean of precision and recall), MCC (describing the correlation coefficient between actual classification and predicted classification),AUROC (area under the receiver operating characteristic curve) and AUPRC (area under the precision recall curve, which measures the problem of sample imbalance) perform comprehensive verification and comparison of the model.

The formulas are shown in (1) to (5), where TP, TN, FP and FN represent true positive, true negative, false positive and false negative respectively.

$Accuracy=\frac{TP+TN}{TP+TN+FP+FN}$ (1)

$Precision=\frac{TP}{TP+FP}$ (2)

$Recall=\frac{TP}{TP+FN}$ (3)

$F1=\frac{2*Precision*Recall}{Precision+Recall}$ (4)

$MCC=\frac{TP*TN-FP*FN}{\sqrt{\left( TP+FP \right)*\left( TP+FN \right)*\left( TN+FP \right)*\left( TN+FN \right)}}$ (5)

**Performance effect of the model on parameter Subgraph division radio and EGCL layer number**

Table S3. Performance effect of the model on parameter Subgraph division radio

| Subgraph division radio | ACC | Precision | Recall | F1 | MCC | AUROC | AUPRC |
| --- | --- | --- | --- | --- | --- | --- | --- |
| 0.70 | **0.880** | **0.618** | 0.625 | 0.621 | 0.550 | 0.891 | 0.665 |
| 0.60 | 0.878 | 0.605 | 0.657 | **0.630** | **0.558** | **0.892** | **0.666** |
| 0.50 | 0.875 | 0.599 | 0.632 | 0.615 | 0.541 | 0.889 | 0.654 |
| 0.40 | 0.859 | 0.546 | 0.634 | 0.587 | 0.504 | 0.879 | 0.627 |
| 0.30 | 0.874 | 0.602 | 0.594 | 0.598 | 0.523 | 0.876 | 0.636 |
| 0.20 | 0.870 | 0.592 | 0.564 | 0.577 | 0.501 | 0.870 | 0.615 |
| 0.10 | 0.857 | 0.536 | **0.688** | 0.603 | 0.523 | 0.891 | 0.649 |

Table S4. Performance effect of the model on parameter EGCL layer number

| EGCL layer number | ACC | Precision | Recall | F1 | MCC | AUROC | AUPRC |
| --- | --- | --- | --- | --- | --- | --- | --- |
| 6 | **0.878** | **0.605** | 0.657 | **0.630** | **0.558** | **0.892** | **0.666** |
| 5 | 0.871 | 0.586 | 0.622 | 0.604 | 0.527 | 0.888 | 0.655 |
| 4 | 0.873 | 0.593 | 0.622 | 0.607 | 0.531 | 0.884 | 0.643 |
| 3 | 0.863 | 0.555 | **0.678** | 0.610 | 0.532 | 0.886 | 0.652 |

**Performance comparison of different experiments**


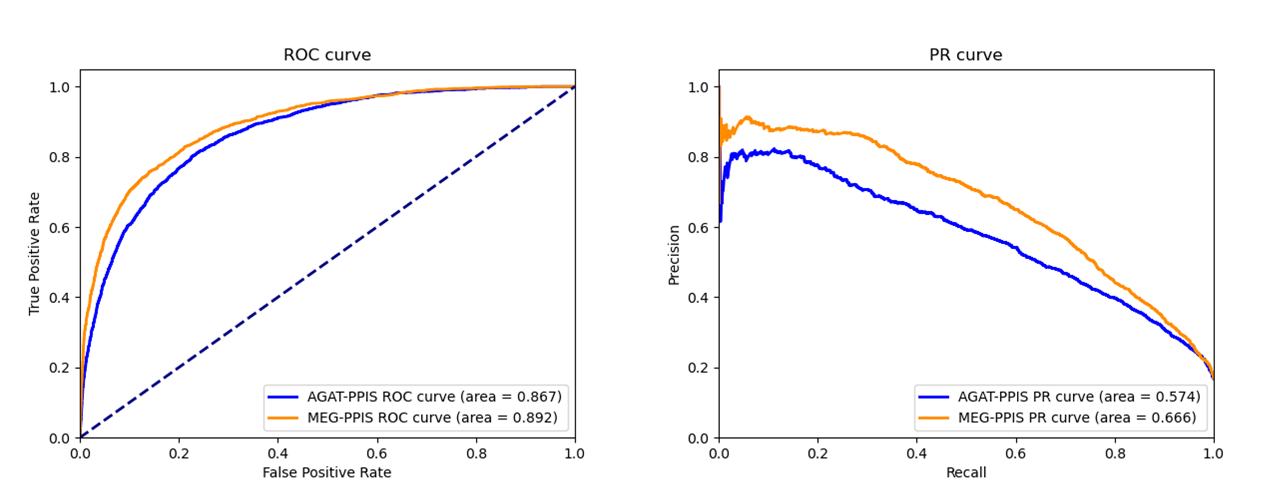


Figure S1. AUROC and AUPRC curve of MEG-PPIS on Test_60

Table S5. Performance comparison of MEG-PPIS with or without subgraph channel on Test_315-28, BTest_31-6 and UBtest_31-6

| Method | Test_315-28 | | BTest_31-6 | | | UBtest_31-6 | | |  |
| --- | --- | --- | --- | --- | --- | --- | --- | --- | --- |
|  | MCC | AUPRC | | MCC | AUPRC | | MCC | AUPRC | |
| MEG-PPIS (without subgraph channel) | 0.530 | 0.613 | | 0.517 | 0.599 | | 0.353 | **0.399** | |
| MEG-PPIS | **0.557** | **0.651** | | **0.583** | **0.641** | | **0.356** | 0.396 | |

**More protein prediction result comparisons about MEG-PPIS and AGAT-PPIS**


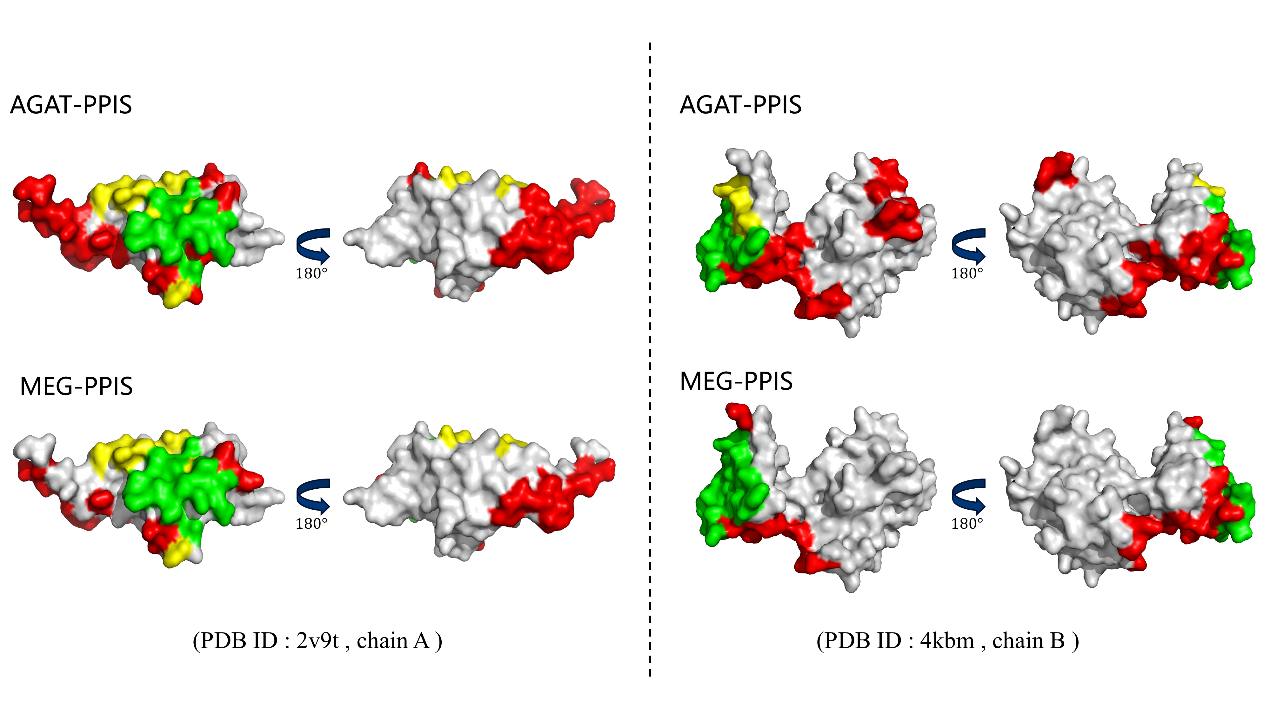


Figure S2. The visualization of the comparison of prediction results between MEG-PPIS and AGAT-PPIS on Specific Proteins


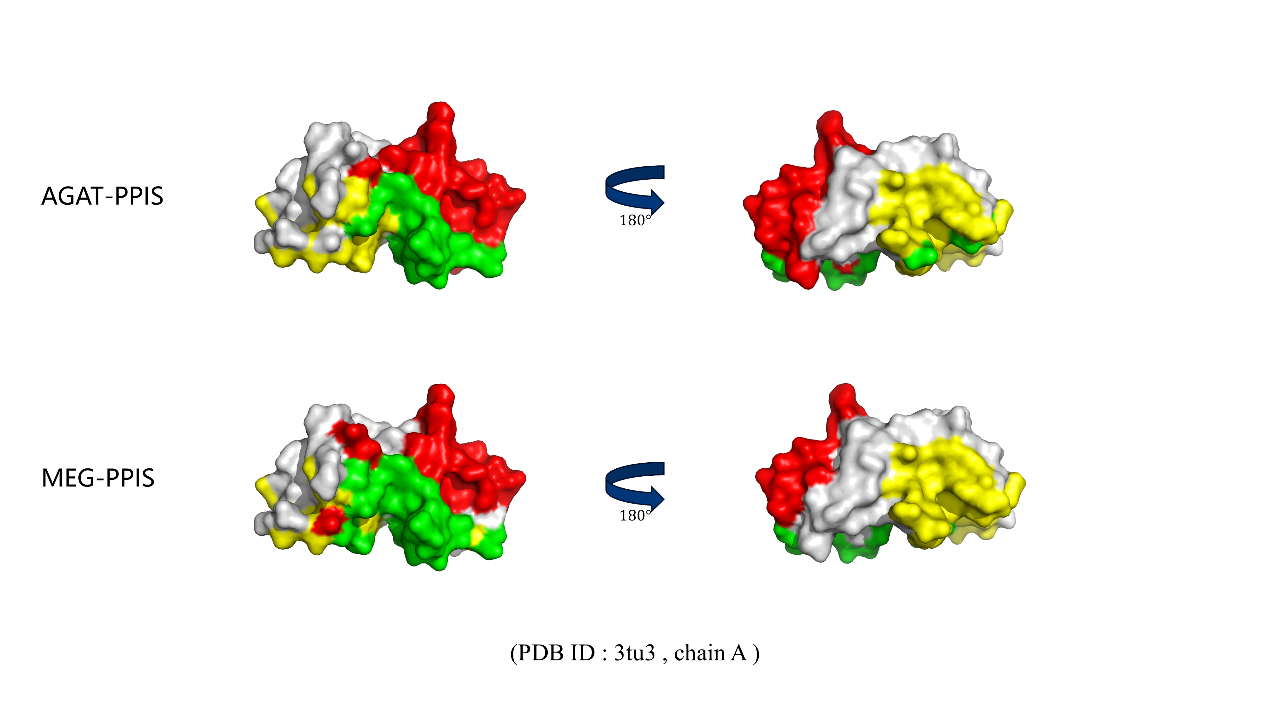


Figure S3. The visualization of the comparison of prediction results between MEG-PPIS and AGAT-PPIS on a Specific Protein (PDB ID: 3tu3, Chain A)

Table S6. Prediction Results of MEG-PPIS and AGAT-PPIS on a Specific Protein (PDB ID: 3tu3, Chain A)

| 3tu3, chain A | TP | TN | FP | FN |
| --- | --- | --- | --- | --- |
| AGAT-PPIS | 19 | 50 | 26 | 24 |
| MEG-PPIS | **20** | **56** | **20** | **23** |


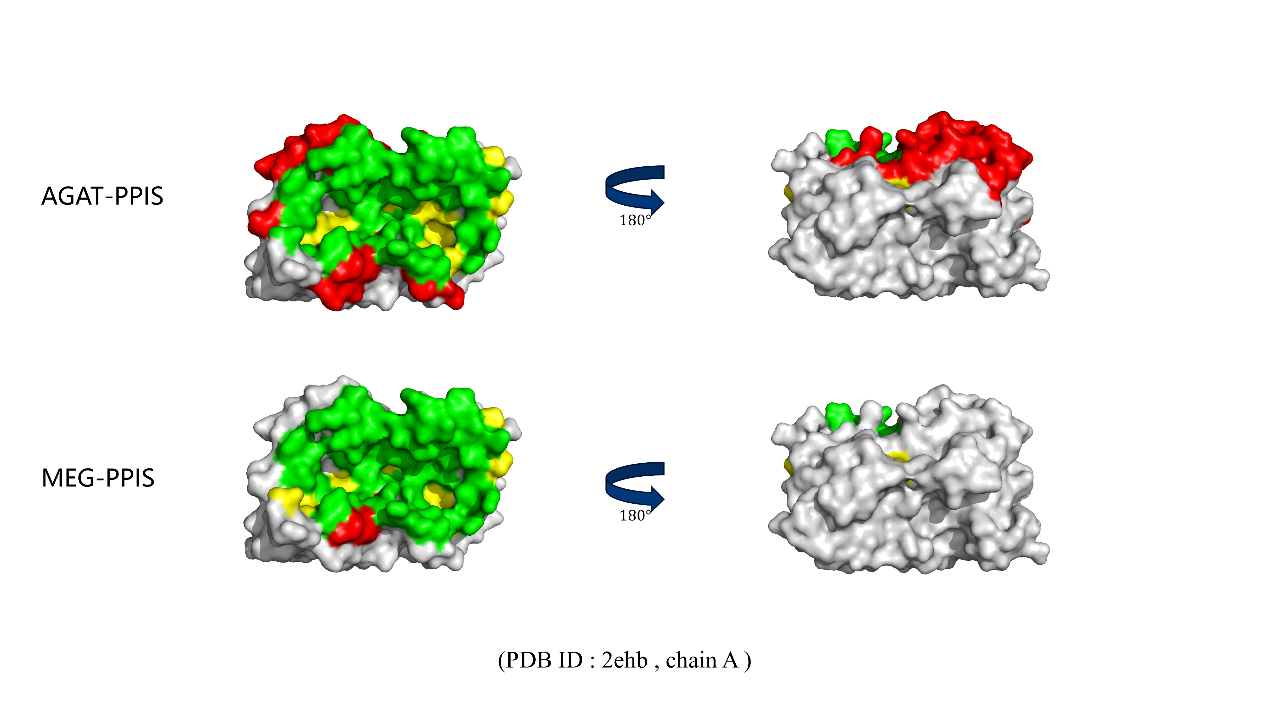


Figure S4. The visualization of the comparison of prediction results between MEG-PPIS and AGAT-PPIS on a Specific Protein (PDB ID: 2ehb, Chain A)

Table S7. Prediction Results of MEG-PPIS and AGAT-PPIS on a Specific Protein (PDB ID: 2ehb, Chain A)

| 2ehb, chain A | TP | TN | FP | FN |
| --- | --- | --- | --- | --- |
| AGAT-PPIS | 40 | 97 | 30 | 15 |
| MEG-PPIS | **44** | **124** | **3** | **11** |


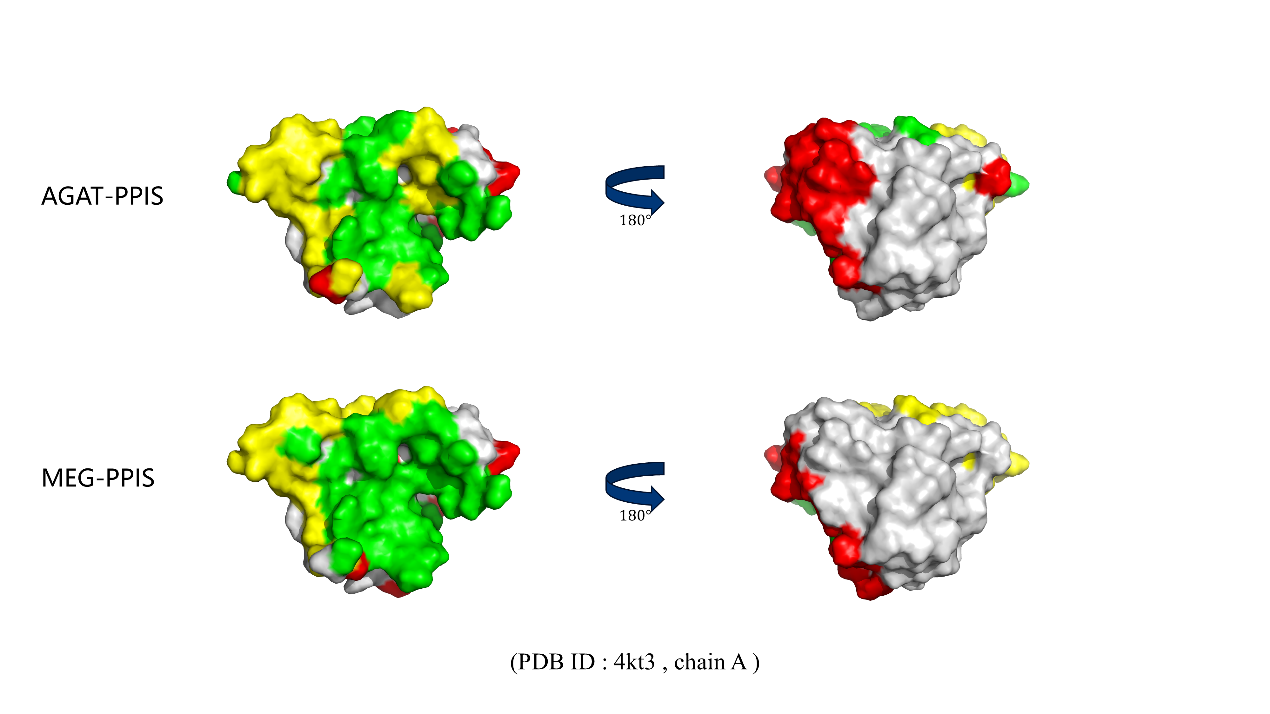
Figure S5. The visualization of the comparison of prediction results between MEG-PPIS and AGAT-PPIS on a Specific Protein (PDB ID: 4kt3, Chain A)

Table S8. Prediction Results of MEG-PPIS and AGAT-PPIS on a Specific Protein (PDB ID: 4kt3, Chain A)

| 4kt3, chain A | TP | TN | FP | FN |
| --- | --- | --- | --- | --- |
| AGAT-PPIS | 19 | 79 | 19 | 21 |
| MEG-PPIS | **26** | **84** | **14** | **14** |
